# Resilience amongst Australian Aboriginal Youth: An Ecological Analysis of Factors Associated with Psychosocial Functioning in High and Low Family Risk Contexts

**DOI:** 10.1371/journal.pone.0102820

**Published:** 2014-07-28

**Authors:** Katrina D. Hopkins, Stephen R. Zubrick, Catherine L. Taylor

**Affiliations:** Telethon Kids Institute, The University of Western Australia, Perth, Western Australia, Australia; Mälardalen University, Sweden

## Abstract

We investigate whether the profile of factors protecting psychosocial functioning of high risk exposed Australian Aboriginal youth are the same as those promoting psychosocial functioning in low risk exposed youth. Data on 1,021 youth aged 12–17 years were drawn from the Western Australian Aboriginal Child Health Survey (WAACHS 2000–2002), a population representative survey of the health and well-being of Aboriginal children, their families and community contexts. A person-centered approach was used to define four groups of youth cross-classified according to level of risk exposure (high/low) and psychosocial functioning (good/poor). Multivariate logistic regression was used to model the influence of individual, family, cultural and community factors on psychosocial outcomes separately for youth in high and low family-risk contexts. Results showed that in high family risk contexts, prosocial friendship and low area-level socioeconomic status uniquely protected psychosocial functioning. However, in low family risk contexts the perception of racism increased the likelihood of poor psychosocial functioning. For youth in both high and low risk contexts, higher self-esteem and self-regulation were associated with good psychosocial functioning although the relationship was non-linear. These findings demonstrate that an empirical resilience framework of analysis can identify potent protective processes operating uniquely in contexts of high risk and is the first to describe distinct profiles of risk, protective and promotive factors within high and low risk exposed Australian Aboriginal youth.

## Introduction

Risks aggregate in low SES environments and the physiological and physical health consequences of multiple and conjoint risk exposure is thought to be one mechanism sustaining socioeconomic disparities in health [1], [2]. The excess burden of risks facing low SES families are further amplified for ethnic minority families exposed to additional constraints on their development such as racism, discrimination, and social and cultural alienation [3]–[6]. Additionally, many Indigenous peoples live with the economic exclusion, family violence and other downstream expressions of the grief and trauma associated with the forcible colonization of their lands, population subjugation and vilification of their societies and culture [7]–[9].

Perhaps nowhere is there greater imperative for understanding the contemporary processes contributing to and perpetuating SES disparities than within Australian Aboriginal and Torres Strait Islander (referred to hereafter as ‘Aboriginal’) children. The Australian Aboriginal population remains disproportionately affected by the multiple risks of poverty. These include a lower rate of high school completion, higher rates of long term unemployment, higher health morbidity and mortality rates, and excessive rates of incarceration relative to the general Australian population [10]. Despite many decades of efforts to address the underlying causes of entrenched disadvantage [11] there has been relatively little substantial movement towards closing gaps between key indicators of Indigenous and non-Indigenous socioeconomic wellbeing [10], [12]. Addressing these socioeconomic and health disparities remains an urgent priority for Australian governments and communities [13]–[15].

It is in this current circumstance of prolonged and arguably limited progress in risk mitigation, that resilience methodologies may offer a complementary perspective to inform government and community strategies designed to address these disparities.

### Resources, risks and psychosocial functioning within the Australian Aboriginal population

In Western Australia, a population representative survey of 0–17 year old Aboriginal children and their families, the Western Australian Aboriginal Child Health Survey (WAACHS 2000–2002, http://aboriginal.telethonkids.org.au/) provides a rare opportunity to explore the relationships between psychosocial risks and resilience amongst Aboriginal youth.

Overall, the WAACHS found a significantly higher percentage of Aboriginal children aged 4–17 years were at high risk of clinically significant emotional or behavioral difficulties (i.e. poor psychosocial functioning) compared to non-Aboriginal children, (24% and 15% respectively), with strong positive associations between exposure to high levels of life stress events and reported poor psychosocial functioning [16], [17]. Further, Aboriginal students assessed by their teachers to be at high risk of poor psychosocial functioning were significantly more likely to be absent from school for 26 days or more per year [18]. Yet it is also apparent in these data that not all Aboriginal youth exposed to high life stress events experience poor mental health outcomes. For example, despite significant and strong associations between exposure to life stress events and reported psychosocial difficulties, 57% of the Aboriginal youth in families reporting 7+ life stress events in the previous 12 months were nevertheless found to be at low risk of psychosocial difficulties [16].

Building on the extant resilience literature [19], [20] Hopkins et al [21] identified five specific family-level risks to the psychosocial functioning of Aboriginal youth: 1) sole parent family status, 2) unemployed household, 3) youth-reported harsh parenting, 4) low reported nurturing parenting, and 5) exposure to violence. In the context of high family-level risk exposure (i.e., two or more of the five risks) young people with a prosocial friend were nearly four times as likely as those without (OR 3.68, p = 0.020, 95% CI 1.30, 10.70) to have good psychosocial functioning [22]. However, and more unexpectedly, living in a higher socioeconomically ranked neighborhood and having higher levels of cultural Indigenous knowledge were each independently associated with poorer psychosocial functioning. These latter findings provide some evidence of the risks associated with upward socioeconomic mobility and they encourage further examination of the processes through which, and for whom, cultural knowledge confers risk or resilience.

This work leaves unknown the extent to which these factors represent a unique profile of protection or risk, or whether a different set of factors promote psychosocial functioning for low risk exposed Aboriginal youth. Indeed, resilience research is characterized by its approach to identifying factors and processes that are protective *uniquely* in high risk circumstances, with negligible or no influence in low risk contexts [23]. For example, it is feasible that cultural knowledge, while found to be a risk factor for high risk exposed Aboriginal youth, may nevertheless promote psychosocial functioning in the context of supportive and well-functioning family environments. Separately analyzing ecological influences on psychosocial functioning of youth in high and low family risk settings may reveal different profiles of processes that are not apparent in the analysis of aggregated samples [24]. So, if different profiles of factors are found to protect the development of high risk exposed Aboriginal youth, this may have important practical implications for interventions currently, a) based on the assumption of homogeneity of disadvantage and dysfunction within the Aboriginal population, or b) based on research with general populations. Indeed, it has been argued that stressed and non-stressed population differ in terms of their experiences with and responses to specific risk exposures, and that understanding processes contributing to resilient outcomes in contexts of high risk complements our understanding of normative child development [25], [26].

### Research aims

The aim of this current research is to identify the factors that uniquely protect psychosocial development within high and low family-risk exposed 12–17 year old Western Australian Aboriginal youth. We model the independent influence of individual, peer, family, neighborhood and cultural factors on psychosocial functioning, *separately*, for youth in high and low-family risk contexts. The reasoning for this is both theoretical and practical. Theoretically, we embrace bioecological perspectives which view child development as occurring within multiple domains of influence over time [27], [28] and resilience perspectives highlighting the great variation in individual responses to similar risk exposures [29]. Practically, although the Australian Aboriginal population may be disadvantaged relative to the wider Australian population across a number of socioeconomic dimensions, there is great heterogeneity in the contemporary lived experiences of the Western Australian Aboriginal population [30]. Therefore, our focus is on modelling predictors of positive psychosocial function separately for Aboriginal youth *within* high and low family-risk contexts.

This study contributes to the existing resilience literature by expanding the scant empirical literature on Australian Aboriginal adolescent psychosocial functioning. A recent review of empirical studies of Australian Aboriginal child health found less than 3% had a mental health focus [31]. This current study has an applied focus with the identification of specific factors having direct and important implications for government service delivery and development of targeted interventions for high risk Aboriginal youth.

## Method

As the current study draws data from the WAACHS a brief overview of this representative survey is provided. The WAACHS is an epidemiological survey of 5,289 Aboriginal children aged 0–17 years, and their primary and secondary carers, randomly selected from the state of Western Australia. The survey aims were broadly to identify and describe risk and protective factors associated with children's physical and mental health, educational participation and attainment, and factors building the capacities of communities with Aboriginal children.

The WAACHS implemented a state-wide area-based clustered multi-stage random sample design. The primary sampling unit for the WAACHS was families. Families were eligible to be selected for participation if they had (an) Aboriginal child(ren) aged 0–17 years. When families had more than one Aboriginal child in this age range all Aboriginal children were selected. The WAACHS identified a random sample of 2,386 families with 6,209 children as eligible. Of this, 1,999 (84%) families agreed to participate. These 1,999 families had 5,513 children of whom 1,480 were youth aged 12–17 years. Parent-report data were secured on 1,480 youth and of these youth 1,073 (73%) provided self-report data.

Data on psychosocial functioning of 12–17 year old young people were collected from primary carers and from youth themselves using Goodman's Strengths and Difficulties Questionnaire [32]. Pilot testing and technical analyses revealed overall very good reliability and validity, with subscales achieving alphas of >.70, with the exception of the peer subscale which was not included in the total SDQ score [33]. Three response categories were created from summed scores of the YSR SDQ: normal (0–15), borderline (16–19) or abnormal functioning (20–40). Non-response analysis indicated that young people who did not complete a YSR were more likely to have borderline/abnormal psychosocial functioning (24.2%, 95% CI 17.7, 32.1) compared to those for whom there was a primary carer questionnaire and YSR completed (19.2%, 95% CI 16.2, 22.5), and more likely to have had contact with the police, juvenile justice officers or the children's court [16], therefore under-representing youth at highest risk of poor psychosocial functioning.

Data were weighted to permit estimation of the responses expected from the total population, with 95% confidence. Using weighted population estimates a sample of 1,073 can be extrapolated to an estimated population of 9,100 with 95% confidence that the actual population estimate lays between 9,050 and 9,100. An adapted version of Probability Weighted Iterative Generalised Least Squares technique was used for the WAACHS data to account for the complex survey design of children within families within communities using SAS software [33].

Details of the design and implementation of the WAACHS are described extensively elsewhere (http://aboriginal.telethonkids.org.au/) [30], [34].

### The Current Study

Human Research Ethics approval for this study was obtained from The University of Western Australia (RA/4/1/4810) and the Western Australian Aboriginal Health Information and Ethics Committee (Ref 298–07.10). In addition a regular program of review was undertaken through the Western Australian Aboriginal Consultative Council Advising Research and Evaluation (ACCARE) at the Telethon Kids Institute. The written permission of primary carers or guardians of Aboriginal children aged 0–17 years was obtained for information to be collected on 0–17 year olds, and for youth aged 12–17 years to be interviewed, for the Western Australian Aboriginal Child Health Survey (2000–2002).

### Participants

From the WAACHS sample of 12–17 year old Aboriginal youth, a subset of 1,021 youth (population estimate 8610, 95% CI 8560, 8610) with *both* primary carer response forms and a YSR questionnaire and valid responses to questionnaire items used in this study were considered in scope. Of these 1,021 youth 50% (95% CI 46, 55) were male, and 36% (95% CI 32, 41) were aged 12–13 years, 31% (95% CI 27, 35) 14–15 years, and 33% (95% CI 28, 37) 16–17 years. Comparisons showed no significant differences between this sample and the 1,073 youth on sex, age, or groupings of resilient psychosocial status (described in the next section). Population estimates at the 95% confidence level are reported.

### Measures Dependent variable

#### Resilient Psychosocial status

This derived variable measures psychosocial functioning relative to risk exposure. It is constructed by cross-classifying SDQ scores (described previously) with exposure to family-level risks predictive of poor psychosocial functioning [21]. SDQ scores 0–15 (normal) represent good, and scores 16–40 (borderline/abnormal) represent poor psychosocial functioning. Family risk exposure is a summed score of exposure to five single risks to psychosocial functioning: youth self-reported harsh parenting, low nurturing parenting, and exposure to violence in the last 6 months, and living in a sole parent, unemployed household [21]. A binary variable was created where low family risk  = 0–1 risk factor, and high family risk  = 2 or more risk factors. Cross-classification results in four groups of youth: 1) Expected Good (low risk/good outcome), 2) Vulnerable (low risk/poor outcome), 3) Resilient (high risk/good outcome), and 4) Less Resilient (high risk/poor outcome).

### Independent Variables

#### Individual level - Sex and age of the young person

Youth *sex* (male/female) and *age* are reported (1 = 12–13 years, 2 = 14–15 years, and 3 = 16–17 years).

*Self-esteem* was measured by youth self-reports on six items specifically designed for the target population of Aboriginal youth and rated on a 5-point Likert scale with higher scores indicating higher self-esteem (α = .79, mean = 23.2, SD 4.5). Sample items include: “I feel proud of how I am”, “I can make good things happen for me”, “No matter how bad I feel I know that I will feel better eventually”. These are fully described elsewhere [16, p.608]. High scores indicate higher self-esteem and quartiles are used in logistic regression modeling and continuous scores used for correlation analyses.

*Self-regulation* is indicated by youth self-reports to one item, an ordinal variable asking youth how often, in the past 6 months, they had being involved in physical fights, from 1 =  *never* to 5 = *6 or more times*.

*Perceived racism* is a youth self-reported response (1 =  *no*, 2 =  *yes*) to a single item asking whether, “in the past 6 months, have you ever been treated badly or refused service because you are Aboriginal?”

#### Family level

*Primary carer level of formal education* was measured on an ordinal scale from primary carer responses and recoded for this study to maximize cell sizes, where 1 =  *less than 9 years*, 2 = *10–12 years*, and 3 = *13 or more years*.

*Financial strain* was assessed from an ordinal scale designed for the WAACHS to measure the family's money situation, where primary carers respond on a 5-point scale from 1 =  *spending more money than we get* to 5 =  *can save a lot*. Higher scores are associated with lower levels of family financial strain.

*Life stress events* is a summed measure of the number of life stress events (0–14) occurring in the previous 12 months as reported by the primary carer. A binary measure was used for logistic regression modeling where 1 = *0–6* and 2 = *7–14* life stress events and the continuous measure was used in correlation analyses. *Overuse of alcohol causes problems at home* is a further indicator of stress in the family and youth responded to a single item of whether or not alcohol causes problems at home (1 =  *no*, 2 =  *yes*).

*Parents affected by forced separations* is assessed from questions asked of primary and secondary carers about whether or not they had been affected by government policies of forced separation from their families where 1 =  *neither parent removed* and 2 =  *one or both removed*, and 99 =  *unknown or not applicable*.

#### Culture and neighborhood level

Cultural factors were included under community influences as cultural knowledge and language was associated with living in regions of increasing levels of isolation [16].

*Youth cultural knowledge* assess youth self-reports of cultural knowledge where 1 =  *very little*, 2 =  *some*, and 3 =  *quite a lot/very much*. *Speaks an Aboriginal language* is a youth self-reported measure of the extent of their conversational knowledge of Aboriginal language where 1 =  *none*, 2 = *a few words*, 3 = *a conversation*. *Importance of ceremonial business* is measured from primary carer reports of the extent to which ceremonial business is important, as some research suggests that parental cultural values can influence adolescents' perceptions of discrimination and psychosocial functioning [35]. This variable was coded 1 =  *important*, 2 =  *not important*, and 3 =  *not relevant*.

*Prosocial friendship* is a variable derived from the YSR responses, where 1 =  *No special friend or close mate*, 2 = *low prosocial special friend*, and 3 =  *high prosocial special friend*. This variable was derived from two questions which asked first whether young people had a “special friend or a really close mate” (where 1 =  *no* and 2 =  *yes*). Youth indicating they had a special friend or close mate then rated a further 8 items according to the extent of their friend's prosocial activities. These items included: “takes an active part in school/community sports, clubs or activities”, “uses drugs other than alcohol” (reverse coded), “gets drunk” (reverse coded), “likes to spend lots of time with his/her own family”, “gets into fights” (reverse coded), “goes to church”, “gets into trouble with police” (reverse coded), and “supports and encourages you”. Responses were recorded as 1 =  no, 2 =  yes. These scores were summed and a binary variable created around a mean score split, with scores 9–14 =  *low* and 15+ =  *high prosocial friend*.

*Socio-Economic Index for Areas (SEIFA)* is a geographic measure of socioeconomic disadvantage calculated from census data and indexing relative socio-economic disadvantage for each census district in Australia [36]. As the majority of Aboriginal children live in families in the bottom 50% of SEIFA, area rankings were grouped into a three-part variable to maximize cell sizes and facilitate logistic regression modeling where 1 =  *bottom 10%*, 2 = *10–50%* and 3 =  *highest 50%* of socioeconomically advantaged areas.

### Data analysis

The WAACHS sample was selected in three stages: census collection districts (CDs), families, and children. CDs were selected with the probability of inclusion proportional to the number of Aboriginal and Torres Strait Islander children living in the CD. As a result, hierarchical logistic regression modeling was used to account for the nested structure of the survey data. Simultaneous multivariate logistic regression modeling was used to compare the differential independent influence of predictor variables on psychosocial functioning for youth separately in contexts of high (Model 1) and low (Model 2) family risk. Thus in Model 1 the independent effects of predictor variables were assessed on the likelihood of Resilient (vs. Less Resilient) psychosocial status, and in low risk exposed Model 2, the likelihood of Expected Good functioning (vs. Vulnerable) psychosocial status. Logistic regression modeling takes into account the potential multiple inter-relationships between predictor variables and determines the effect of each predictor variable on the outcome variable *independent* of the effect of all other variables in the model. The same set of predictor variables were entered simultaneously in each model as this method is appropriate when no a priori hypotheses are made about respective order of importance of predictor variables [37]. SAS version 9.2 was used for all analyses (SAS Institute Inc., Cary, NC, USA, 2000–2008).

Reported associations between the outcome variables and the predictor variables are expressed as odds ratios. Odds ratios of less than 1.0 denote a reduced likelihood of positive psychosocial functioning relative to the reference category, and odds ratios of greater than 1.0 an increased likelihood of positive psychosocial functioning relative to the reference category [38]. The goodness-of-fit of each model was assessed by convergence being achieved using Predicted Quasi-Likelihood Estimation, and model statistics (parameter estimates, standard errors, degrees of freedom, t-values, probabilities and 95% confidence intervals).

Model convergence was initially not achieved for the Expected Good vs. Vulnerable model when measures of primary carer level of education and family financial strain were included, potentially due to low cell numbers in the Vulnerable group. As convergence was achieved either including or excluding these same variables in the Resilient vs. Less Resilient model without significantly affecting the results they were removed from both models to achieve convergence and retain equivalence.

## Results

Bivariate relationships between resilient psychosocial status and each of the five single risks comprising Family-level risk exposure were examined for significant differences within high and low risk exposed groups (Table 1). With the exception of harsh parenting in high risk contexts (Resilient and Less Resilient groups) each single risk measure was experienced relatively equally (as indicated by overlapping CIs) within each group. Indeed, a higher proportion of Resilient (62.3%) compared to Less Resilient youth (45.7%) reported harsh parenting. Exposure to family violence was the single most frequently reported risk, experienced by 37% (95% CI 31, 42.5) of youth in Expected Good families to 92% (95% CI 86.3, 95.5) of Less Resilient youth. Table 1 shows 14.3% (95% CI 12.0, 17.0) of youth experienced no family-level risk factors, 36.9% (95% CI 33.6, 40.4) had one risk, 31.3% (95% CI 28.1, 34.6) had two risks, 13.9% (95% CI 11.6, 16.7) had 3 risks, 3.3% (95% CI 2.4, 4.4) had 4 risks, and 0.3% (95% CI 0.0, 2.9) had 5 risks. As a binary measure 50.7% (95% CI 47.0, 54.4) of youth experienced 0–1 risks and the remainder 2+ risks. The majority of youth (68.5%, 95% CI 65.1, 71.6) had normal levels of psychosocial functioning.

**Table 1:** Psychosocial functioning mean score and percentage of family-level risk exposure by Psychosocial Resilient Status, 12–17 year-old Aboriginal youth (n = 8610, 95% CI 8560, 8610).

*Variable*	*Expected Good*	*Vulnerable*	*Resilient*	*Less Resilient*
Psychosocial functioning: Mean SDQ score (95% CI)	10 (10, 10)	19 (18, 19)	11 (10, 11)	19 (19, 20)
Family-level risk (%, 95% CI) -				
None	30.8 (25.6, 36.3)	17.2 (9.5, 26.7)	-	-
Single headed household	15 (11.8, 20.7)	20 (12.9, 28.5)	51.0 (43.5, 58.1)	54.6 (46.9, 62)
Unemployed	2.2 (0.9, 5.2)	2.6 (0.4, 11)	16.8 (11.7, 23.7)	14.3 (7.6, 22.5)
Harsh parenting	4.4 (2.4, 7.1)	3.7 (1.1, 10.1)	62.3 (55.2, 68.9)	45.7 (38.6, 53.2)
Low nurturing parenting	9.8 (7.1, 13.1)	7.9 (3.7, 15.8)	41.6 (34.4, 48.7)	50.2 (42.2, 57.8)
Exposed to family violence	36.8 (31, 42.5)	51.2 (40.4, 61.7)	89.7 (84.3, 94.2)	92.0 (86.3, 95.5)
**Total (95% CI)**	**3420 (3100, 3740)**	**950 (770, 1140)**	**2480 (2200, 2770)**	**1770 (1540, 2030)**

The person-based classification of psychosocial resilient status reveals over one-third of all youth have Expected Good outcomes (i.e., low family level risk/good psychosocial functioning, 39.7%, 95% CI 36, 43.3), with over one-quarter Resilient (i.e., high family level risk/good psychosocial functioning, 28.8%, 95% CI 25.6, 32.2). Of those youth living in high family level risk contexts more than half (58.4%, 95% CI 53.8, 63.1) were identified as Resilient.

Details of bivariate relations between predictor variables and resilient psychosocial status groups are shown below in Table 2.

**Table 2:** Sociodemographic characteristics of Aboriginal youth 12–17 years, by psychosocial resilient classification (n = 8610, 95% CI 8560, 8610).

		Expected Good n = 3420 (3100, 3740)	Vulnerable n = 950 (770, 1140)	Resilient n = 2480 (2200, 2770)	Less Resilient n = 1770 (1540, 2030)
*Variable*		*% (95% CI)* 39.7 (36, 43.3)	*% (95% CI)* 11.0 (9, 13.3)	*% (95% CI)* 28.8 (25.6, 32.2)	*% (95% CI)* 20.5 (17.8, 23.5)
*Individual*					
% Males		54 (48, 59.8)	52.7 (41.1, 63)	49.7 (42.2, 56.7)	44.5 (37.4, 51.7)
Age group	12–13 years	34.4 (29.0, 39.9)	36.2 (27.5, 45.4)	43.4 (36.3, 50.4)	39.7 (32.4, 47.1)
	14–15 years	35.3 (29.6, 41.4)	30.3 (22, 39.4)	30.6 (24.6, 36.8)	37.9 (30.7, 45.4)
	16–17 years	30.3 (25.3, 36.1)	33.5 (24.9, 42.6)	26.0 (20.5, 32.3)	22.3 (17.1, 28.1)
Never been in a fight		71.2 (65.5, 76.8)	47.2 (37.2, 57.2)	58.6 (51.5, 65.2)	45.2 (38.3, 52.4)
Perceived racism		13.8 (10, 18.6)	26.9 (19.8, 35.3)	19.9 (14.8, 25.6)	33.1 (25.2, 41.3)
Self-esteem	Highest quartile	31.6 (26.6, 37.2)	29.4 (22.0, 38.7)	25.4 (19.9, 31.4)	15.9 (11.2, 21.2)
	Lowest quartile	16.9 (12.9, 21.3)	26.6 (19.5, 35.6)	28.5 (22.1, 35.1)	42.8 (35.8, 50.3)
*Family*					
Primary carer education	9 years or less	32.3 (26.6, 38l9)	24.1 (16.0, 33.1)	31.2 (25.0, 38.5)	29.9 (23.4, 36.9)
	10–12 years	58.9 (52.4, 65.0)	71.1 (61.5, 79.2)	60.6 (52.9, 67.9)	63.9 (56.4, 71.3)
	13+ years	8.8 (5.5, 13.1)	4.8 (2.3, 12.3)	8.1 (3.4, 14.6)	6.3 (2.5, 12.3)
Family financial strain	Spending more than we get	8.6 (5.5, 13.0)	8.8 (4.4, 16.1)	11.8 (8.1, 16.8)	8.7 (4.7, 13.9)
	Just enough to get by	41.9 (35.7, 48.1)	41.6 (31.8, 52.6)	51.9 (44.3, 59.6)	51.5 (43.8, 59.0)
	Some leftover but we spend it	13.6 (9.9, 18.2)	16.8 (9.4, 27.5)	11.3 (7.1, 17.1)	14.5 (10.5, 19.1)
	Save a bit	30.5 (24.8, 36.5)	28.4 (19.1, 38.6)	20.0 (14.4, 26.8)	19.5 (13.8, 26.3)
	Save a lot	5.3 (2.8, 8.8)	4.4 (2.5, 7.6)	5.0 (1.4, 16.1)	5.8 (2.8, 11.3)
7–14 Life Stress Events		17.2 (12.8, 22.7)	21.8 (14.9, 30.1)	22.6 (17.4, 29)	26.7 (20.2, 33.7)
No alcohol problems at home		80.7 (75.6, 85.4)	67.9 (58.2, 76.7)	68.4 (62, 74.4)	64.3 (56.8, 71.3)
One or both parents forcibly removed		15.9 (11.8, 20.5)	17.8 (11.7, 25.7)	22.6 (16.5, 29.9)	19.5 (13.5, 26.5)
*Culture*					
Youth reports quite a lot/very much cultural knowledge		33.0 (29.2, 37.2)	36.8 (28.6, 45.6)	23.6 (19.2, 28.6)	29.4 (23.4, 35.9)
Youth can converse in Aboriginal language		15.3 (11.3, 19.7)	24.2 (17.9, 31.5)	13.1 (8.2, 19)	12.2 (8, 17.7)
Carer reports ceremonial business important		63.9 (57.5, 70)	64.5 (52.4, 74.7)	62.9 (55.7, 69.7)	64.9 (57.2, 71.8)
*Neighborhood*					
Prosocial friend		75.1 (69.9, 79.7)	64.2 (53.8, 73.4)	70.0 (62.7, 76.5)	50.4 (43.1, 57.9)
SEIFA – top 50%		35.5 (29.1, 42.2)	38.3 (27.3, 49.2)	36 (28.4, 43.7)	42.8 (34.5, 51.8)

### Model 1 High risk exposure - Modeling likelihood of Resilient vs. Less Resilient status

Using multivariate logistic regression we modeled 13 predictor variables on the likelihood of Resilient compared to Less Resilient group status and found four variables significantly associated with resilient psychosocial functioning: 1) high self-esteem, 2) high self-regulation, 3) having a prosocial friend and 4) living in neighborhoods ranked lower on socioeconomic advantage (see Table 3).

**Table 3:** Modeling the likelihood of Resilient vs. Less Resilient psychosocial functioning by Individual, Family, Culture and Neighborhood characteristics (n = 4250, 95% CI 4200, 4250).

	*Parameter*	*Estimate*	*SE*	*Df*	*T*	*P-value*	*Odds ratio*	*95% CI*
	Intercept	−.75	.63	259	−1.2	.24		
Sex	Male	.38	.23	123	1.7	.10	1.46	.93, 2.29
	Female	0	.	.	.	.	1.00	
Age group	Younger	0	.	.	.	.	1.00	
	Middle	−.11	.27	123	−.42	.68	.89	.52, 1.53
	Older	.48	.30	123	1.62	.11	1.62	.90, 2.91
Self-esteem	Low – 1st quartile	0	.	.	.	.	.	.
	2nd	.30	.34	123	.91	.37	1.36	.70, 2.62
	3rd	.68	.29	123	2.32	.02	1.97	1.11, 3.51
	High – 4th quartile	.70	.34	123	2.03	.05	2.01	1.02, 3.93
In a fight in last 6 months?	Never	0	.	.	.	.	1.00	
	Once	−.60	.26	123	−.2.2	.03	.56	.33, .93
	2–3 times	−.13	.33	123	−.40	.69	.88	.46, 1.67
	4–5 times	−.30	.66	123	−.45	.65	.74	.20, 2.72
	6 or more times	−1.06	.50	123	−2.11	.04	.35	.13, .93
Treated badly because of your race	Yes	0	.	.	.	.	1.00	
	No	.22	.25	123	.86	.39	1.24	.76, 2.03
Primary carer Life Stress Events (no.)	7–14	0	.	.	.	.	1.00	
	0–6	−.08	.25	123	−.30	.76	.93	.57, 1.51
Alcohol causes problems at home-	Yes	0	.	.	.	.	1.00	
	No	.28	.25	123	1.11	.27	1.32	.81, 2.15
One or both parents affected by forced separation—	No	0	.	.	.	.	1.00	
	Yes	.29	.28	123	1.03	.31	1.34	.77, 2.32
	Not known/not applicable	.01	.39	126	.03	.98	1.01	.47, 2.16
Youth cultural knowledge	Very little	0	.	.	.	.	1.00	
	Some	.09	.33	123	.65	.52	1.18	.71, 1.95
	Quite a lot/very much	−.16	.32	123	−.50	.62	.85	.45, 1.60
Youth speaks language	No	0	.	.	.	.	1.00	
	A few words	.14	.28	123	.50	.62	1.15	.66, 1.99
	A conversation	−.16	.37	123	−.43	.67	.86	.42, 1.76
Primary carer importance of ceremonial business	Not important	0	.	.	.	.	1.00	
	Important	−.19	.31	123	−.60	.55	.83	.45, 1.52
	Not relevant	.16	.34	126	.47	.64	1.17	.61, 2.27
Prosocial Special Friend	No special friend	0	.	.	.	.	1.00	
	Low Prosocial friend	−.06	.42	123	−.14	.89	.94	.41, 2.17
	High Prosocial friend	.93	.40	123	2.33	.02	2.52	1.16, 5.49
Neighborhood SEIFA	Bottom 10%	0	.	.	.	.	1.00	
	10%–50%	−.40	.24	259	−1.67	.10	.67	.42, 1.07
	Highest 50%	−.89	.44	259	−2.01	.05	.41	.17, .98

Note. SE = standard error; Df = degrees of freedom; T = t value; P = probability value; Odds Ratio 95% CI =  confidence interval, lower limit and upper limit.

At the individual level, young people in the upper third or fourth quartiles of self-esteem were nearly twice as likely as those with low self-esteem to be Resilient (p = .02, OR 1.97, 95% CI 1.11, 3.51, and p = .05, OR 2.01, 95% CI 1.02, 3.93 respectively). Young people reporting being in a fight once (p = .03, OR .56, 95% CI .33, .93) or 6 or more times (p = .04, OR .35, 95% CI .13, .93) were less likely to be Resilient than youth reporting never fighting in the previous 6 months. At the neighborhood level young people with a prosocial friend were two and a half times more likely to be Resilient than young people with no special friend (p = .02, OR 2.52, 95% CI 1.16, 5.49), however living in neighborhoods ranked in the highest 50% of socioeconomic advantage was associated with lower likelihood of Resilient functioning (p = .05, OR .41, 95% CI .17, .98). None of the family level variables measured in this study (life stress events, alcohol not causing problems at home, or parents affected by forced separations), were significantly associated with the likelihood of Resilient vs. Less Resilient status in the context of high family-level contextual risk.

### Model 2 Low risk exposure - Modeling likelihood of Expected Good vs. Vulnerable status

The likelihood of Expected Good vs. Vulnerable status was modeled next using the same 13 predictor variables. Three variables were found to be significantly associated with increased likelihood of Expected Good vs. Vulnerable status, higher self-esteem and self-regulation (getting into less fights), and not being exposed to racism (see Table 4).

**Table 4:** Modeling the likelihood of Expected Good vs. Vulnerable psychosocial functioning by Individual, Family, Culture and Neighborhood characteristics (n = 4360, 95% CI 4310, 4360).

	*Parameter*	*Estimate*	*SE*	*Df*	*T*	*P*	*Odds ratio*	*95% CI*
	Intercept	.54	.87	265	.61	.54		
Sex	Female	0	.	.	.	.	1.00	
	Male	.33	.26	112	1.28	.20	1.39	.84, 2.31
Age group	Younger	0	.	.	.	.	1.00	
	Middle	.28	.29	112	.95	.34	1.32	.75, 2.33
	Older	.06	.37	112	.16	.87	1.06	.51, 2.19
Self-esteem	Low – 1st quartile	0	.	.	.	.	1.00	
	2nd	−.10	.39	112	−.26	.80	.90	.42, 1.95
	3rd	.90	.38	112	2.36	.020	2.46	1.16, 5.18
	High – 4th quartile	.29	.36	112	.81	.41	1.34	.66, 2.70
In a fight in last 6 months?	Never	0	.	.	.	.	1.00	
	Once	−1.00	.31	112	−3.18	.002	.37	.20, .68
	2–3 times	−1.23	.40	112	−3.07	.003	.29	.13, .64
	4–5 times	−2.22	.78	112	−2.84	.005	.11	.02, .50
	6 or more times	−.97	.52	112	−1.86	.07	.38	.14, 1.05
Treated badly because of your race	Yes	0	.	.	.	.	1.00	
	No	.74	.30	112	2.48	.015	2.09	1.17, 3.74
Primary carer Life Stress Events (no.)	7–14	0	.	.	.	.	1.00	
	0–6	−.06	.31	112	−.20	.84	.94	.51, 1.72
Alcohol causes problems at home-	Yes	0	.	.	.	.	1.00	
	No	.51	.31	112	1.65	.10	1.66	.91, 3.03
One or both parents affected by forced separation	No	0	.	.	.	.	1.00	
	Yes	−.13	.35	112	−.21	.83	1.14	.58, 2.25
	Not known/not applicable	.43	.45	130	.97	.33	1.54	.64, 3.70
Youth cultural knowledge	Very little	0	.	.	.	.	1.00	
	Some	.09	.33	112	.28	.78	1.10	.58, 2.09
	Quite a lot/very much	.15	.33	112	.46	.65	1.16	.61, 2.23
Youth speaks language	No	0	.	.	.	.	1.00	
	A few words	−.09	.33	112	−.27	.79	.91	.48, 1.75
	A conversation	−.74	.46	112	−1.59	.12	.48	.19, 1.19
Primary carer importance of ceremonial business	Not important	0	.	.	.	.	1.00	
	Important	−.00	.32	103	−.00	1.00	1.00	.53, 1.87
	Not relevant	.52	.49	104	1.06	.29	1.68	.64, 4.37
Prosocial Special Friend	No special friend	0	.	.	.	.	1.00	
	Low Prosocial friend	−.22	.53	112	−.43	.67	.80	.28, 2.25
	High Prosocial friend	.08	.49	112	.16	.87	1.08	.42, 2.81
Neighborhood SEIFA	Bottom 10%	0	.	.	.	.	1.00	
	10%–50%	−.37	.38	265	−1.08	.28	.69	.36, 1.34
	Highest 50%	−.45	.37	265	−1.22	.22	.64	.31, 1.31

Note. SE = standard error; Df = degrees of freedom; T = t value; P = probability value; Odds Ratio 95% CI =  confidence interval, lower limit and upper limit.

Young people in the third quartile but not the fourth (highest) quartile of self-esteem were two and a half times as likely as those in the lowest quartile of self-esteem to be in the Expected Good group (p = .02, OR 2.46, 95% CI 1.16, 5.18). Youth reporting being in a fight once (p = .002, OR .37, 95% CI .20, .68), 2–3 times (p = .003, OR .29, 95% CI .13, .64), or 4–5 times (p = .005, OR .11, 95% CI .02, .50) in the last 6 months were significantly less likely than those reporting never getting in to fights to be in the Expected Good group. Finally, young people reporting no exposure to racism were more than twice as likely (p = .015, OR 2.09, 95% CI 1.17, 3.74) as those who did report exposure to racism to be in the Expected Good group. No significant associations were found for measures of stressful life events, alcohol causing problems at home and cultural connection variables.

### Factors uniquely associated with positive psychosocial functioning in high family-risk contexts

Models 1 and 2 were then compared to identify variables significant in high but not low family-level risk context. Two variables were uniquely associated with good psychosocial functioning in high risk contexts: prosocial friendship and living in low socioeconomic neighborhoods. For youth in high family-level risk contexts, having a prosocial friend conferred unique protection (p = .02, OR 2.57, 95% CI 1.17, 5.64), and living in more socioeconomically advantaged neighborhoods conferred additional risk. Relative to youth in the lowest 10% of neighborhoods ranked by socioeconomic advantage, those youth in the highest 50% of neighborhoods were less likely to be Resilient (p = .041, OR .42, 95% CI .18, .96).

In low family-risk contexts youth only one factor was uniquely associated with good psychosocial functioning. Youth reporting not being exposed to racism were more than twice as likely as those exposed to racism to have good psychosocial functioning (p = .02, OR 2.09, 95% CI 1.17, 3.74).

Finally, two factors at the individual level were identified as generally beneficial for Aboriginal youth in both high and low family risk exposed contexts. Higher levels of self-esteem and self-regulation (no reported fighting in the last 6 months) were significantly associated with normal psychosocial functioning for both Resilient (high family risk) and Expected Good (low family risk) youth.

## Discussion

These findings extend our previous research by estimating associations of psychosocial resilience in a large sample of Australian Aboriginal youth and identifying access to social resources as uniquely protecting psychosocial functioning of high risk exposed youth. Over one-half (58%) of youth living in high family-level risk contexts predictive of poor psychosocial functioning were defined as Resilient. The profiles of factors uniquely associated with positive psychosocial function in high risk exposed but Resilient youth are discussed next, before discussing those operating uniquely for youth in low risk contexts, and finally a comment on those factors failing to reach significance.

### Protective factors operating uniquely in high risk contexts

#### Prosocial friendship

In the context of high family risk Aboriginal young people with a prosocial friend were more than twice as likely to have positive psychosocial functioning as those youth reporting no special friend. The positive influence of prosocial friendship on adaptive functioning is consistent with studies of the positive adaptation of maltreated children [39], the CIET studies of resilience in First Nations youth [40], and has been found to moderate the negative impact of perceived racism on health [41]. In the presence of high family risk environments having a prosocial friend is likely to have a positive influence on young people's psychosocial functioning through (a) mediating and moderating the high-risk home environment via provision of social and emotional support, encouragement to engage in health promoting behaviors, and development of coping skills [42]; and (b) enabling opportunities for the at-risk young person to maintain or connect to important extended relationships and interact with other potential role models via their prosocial friend's family, friends and other social networks [40], [43], [44].

The significance of having a prosocial friend as a unique protective factor in high risk contexts has a bearing on the formulation of public policies for community and youth-led initiatives that support and engage vulnerable young people. For example, there is evidence that natural resource management activities, which develop constructive and supportive relationships through land management and conservation projects, offer opportunities for leveraging multiple benefits from existing programs [45], [46]. Increasing fiscal constraints demand more effective use of public resources. Programs such as the bush ranger or junior ranger cadet programs are examples that stand ready to meet both current environmental and land management priorities as well as the needs of Aboriginal youth in high risk families, by a) facilitating prosocial peer relationships and positive adult role models; b) building positive cultural and social connections for Aboriginal youth through the involvement of Elders in natural resource management (NRM) knowledge transfer; c) engaging Aboriginal children and youth in school-based learning linked to NRM objectives; d) providing pathways from school to further training, leading in turn to employment opportunities, particularly in regional and remote parts of Australia where it is difficult to attract permanent employees; and e) potentially delivering improvements in physical, social and emotional wellbeing to individuals, families and communities [47], [48].

#### High neighborhood socioeconomic disadvantage

This current study identified that living in a higher socioeconomically advantaged neighborhood increased the risk of poor psychosocial functioning for youth living in high risk families. This suggests an additional and unique vulnerability factor to their already high risk status which was not significant for low family-level risk youth.

At first glance this appears counterintuitive to evidence of the beneficial effects of socioeconomically advantaged neighborhoods on mental health [17], [49]. Socioeconomic advancement is a central pillar of Australian government policies for Aboriginal Australians that seek to “close the gap” between human development outcomes for Aboriginal Australians relative to the general population. However, the small total Western Australian Aboriginal population (77,928 or 3.4%) remains substantially economically disadvantaged with the vast proportion (71%) of Aboriginal families in predominantly public supported rental housing [34]. The much smaller proportion of Aboriginal families who have moved out of absolute and relative poverty and into socioeconomically advantaged areas is, admittedly, small and largely “unseen” in Australia. There are likely to be substantial stresses associated with upward mobility and movement into more advantaged areas, with resultant relative isolation from extended family, community and cultural supports for Aboriginal youth in upwardly mobile families. While our findings require replication and extension, they echo the mixed findings from the “Moving to Opportunity” program in the United States where findings for young people in families randomly assigned from disadvantage housing into more favorable circumstances revealed differential impacts with both negative and positive outcomes [50], [51]. Similarly, longitudinal research on family and neighborhood effects on youth delinquency in the US has found that when neighborhood supports are available to meet the emotional and belonging needs of young people this may offset risks conferred by the family environment [52]. Further, for minority populations, neighborhood ethnic composition may offer security from expectations of discrimination and racism [53], [54]. The results are thus consistent with some research that shows the beneficial effects of more advantaged neighborhoods may not apply for minority youth [55] and that there may be stresses associated with upward social mobility [56].

### Factors promoting psychosocial functioning for all youth - Self-esteem and self-regulation

In both high and low family-level risk exposed contexts self-esteem and self-regulation were independently associated with increased likelihood of positive psychosocial functioning. In high family risk contexts young people in the third and fourth (highest) quartile of self-esteem were around twice as likely as those in the lowest quartile of self-esteem to have normal psychosocial functioning with the odds-ratios indicating a linear relationship. For low risk exposed youth however, the relationship was only significant for youth in the third quartile of self-esteem. This suggests that although self-esteem may be generally beneficial, youth at highest risk perhaps have a greater capacity to benefit.

These findings are consistent with studies showing positive self-esteem and good self-regulation to be related to resilient functioning [57], [58] as well as being fundamental capacities promoting positive development in a variety of developmental domains [59], [60]. Importantly, longitudinal evidence supports the occurrence of self-esteem prior to depressive symptoms with low self-esteem influencing depressive symptoms over time [61], [62], and low self-esteem linked to negative behaviors such as aggression and antisocial behaviors [63].

A lack of association between cultural connectedness and psychosocial functioning for youth in both high and low risk family contexts was also identified. There is considerable interplay between processes of self-esteem, self-regulation and identity development (both personal and collective or social identities) and the inhibiting or supportive neighborhood contexts in which they occur. Stereotype threat, the psychosocial processes of anticipating being stereotyped and the stigma that “leads (a group) to be devalued in the eyes of others” [64] p.395 [65], may be developmentally salient and potentially damaging for many Aboriginal adolescents. However, the anticipation of being treated differently because of racial group membership, and the consequences for coping strategies invoked, can depend on a number of factors [66], including appraisal of the relevance of an event to self-identity. In contexts where an event is appraised as a threat to one's identity, coping mechanisms may include “engagement versus disengagement strategies”, reflecting respectively a fight or flight response [64] p.404. Importantly, the construction of a devalued social identity varies across time and cultures and within specific relationships and contexts [64], [67]. It is an important aim for societies to address the risk that racism poses for ethnic/minority populations and to understand for whom and in which contexts (e.g., high/low family risk, neighborhood ethnic/SES composition) processes of stereotype threat are triggered and impact the development of positive identity and self-esteem in children and adolescents.

Self-control, but not cultural identity, mediated the impact of racism on depressive symptoms amongst a sample of Australian Aboriginal adults [68]. Similarly, longitudinal research with North American Indian youth found that self-efficacy beliefs were related to lower depressive symptoms over time, and cultural identity did not moderate self-efficacy [69].

### Self-reported Racism – a specific risk for youth in low family risk contexts

Youth in low family-level risk contexts who reported no exposure to racism were nearly two and a half times more likely to have better psychosocial functioning than those youth reporting exposure to racism. On the face of it this result seems consistent with literature revealing the negative effects of racism on psychosocial functioning [41], [70]–[72]. However exposure to racism had no significant association with psychosocial functioning for youth in high family-level risk contexts. This inconsistent effect between youth in high and low family risk contexts may be explained by a) differences in actual exposure to racism, b) the lower salience of racism as a stressor for high risk exposed youth relative to their overall burden of life stressors, or c) differences between groups in the perception and attribution of events as racist. The perception of discrimination is situationally constructed by the individual and its impact may thus depend on the salience of collective and individual racial identities to the individual at that time and in those specific circumstances [73], [74]. There is much more to be learned about the interactions between self-identity and cultural identity development and factors influencing these processes as they emerge from childhood to adolescence. Many other influences are important yet to date have not been extensively investigated in the Australian context. Factors such as the extent of intra-racial racism, contexts in which racism occurs (urban/rural/remote), the influence of socioeconomic position on intra- and inter-personal racism, the subjective experiences of racism, and coping mechanisms employed, may all influence the impact of racism on young people's psychosocial functioning and about which little is known [75].

### Beyond Empirical Measurement?

It is notable that four variables relating to connectedness to culture (primary carer reports of importance of ceremonial business, primary or secondary carer forcibly removed from family; and young people's self-reported knowledge of culture and language) were not independently associated with psychosocial functioning of young people in either high or low family risk contexts. Cultural knowledge, language and participation in traditional activities have been associated with improved psychosocial functioning [76], [77]. Aspects of culture measured at the community or band level and which include high levels of cultural continuity, self-governance, and speaking of Indigenous languages, have been found to be protective against suicide risk [77], [78]. Aboriginal people in Australia emphasize the fundamental value of cultural connections, identity and language as central processes supporting social and emotional wellbeing [79] [80], [81] and are important foundations for psychological healing of the loss and grief following colonization [82], [83]. The inter-relatedness of culture and language, sense of self, connections to others, inheritance, and friendships with racial identity were described in a qualitative study of Western Australian Aboriginal children [84].

Yet the evidence remains mixed. The protective effects of cultural connections on resilience to criminal offending has been described in the Australian context [44] but other research also with a focus on offending describes aspects of culture that are both protective (family connections) and risk factors (family disputes and fighting) [85]. In the Canadian context the CIET studies found that although reported pride in one's heritage was related to resilience, other measures of culture and spirituality were not [40]. Further, connections between cultural identity and mental health appeared to be moderated by feeling supported and self-esteem [40]. A 3-year longitudinal study of North American Indian youth also found cultural identity did not moderate the relationship between self-efficacy and depressive symptoms [69]. Thus the null empirical findings for the influence of cultural factors may reflect the complexity and phenomenology of the construct and the difficulties of defining, measuring and incorporating this into an empirical ecological framework.

### Limitations

The WAACHS was a large, population representative, cross-sectional survey covering a broad range of factors impacting Aboriginal children's lives including their physical health, social and emotional wellbeing, educational experiences, and community and cultural lives. Along with the limitations of cross-sectional studies, the measures utilized in the WAACHS were necessarily short to reduce respondent fatigue and costs of face to face survey methodologies. Nevertheless, the scale and significance of this representative survey of Aboriginal children and youth presents a unique opportunity to explore patterns and associations amongst the multiple and interrelated risks and protections both unique to the historical and contemporary experiences of Aboriginal youth and those generally applicable to all young people.

## Conclusions

The application of a resilience framework of analysis has demonstrated the influence of some factors that work differently for Aboriginal youth depending on their exposure to contextual risk. Despite similar experiences of high family risk, we identified prosocial friendship and lower socioeconomic ranking of neighborhoods as factors uniquely protecting the psychosocial development of Aboriginal youth. Within an obvious overarching aim of risk amelioration, the identification of uniquely protective factors nevertheless provides a platform for supporting at risk young people now. Facilitating their transition into adulthood represents an investment in early intervention enabling them to become the best parents they can be, to then in turn foster the healthy development of future generations of Aboriginal children. Improved understanding of the factors implicated specifically in protecting psychosocial development for those young people at highest risk holds potential for interrupting negative trajectories of development. A sizeable proportion of Aboriginal youth were identified as living in high family risk contexts and for whom specific personal and community characteristics were significantly associated with their relatively positive psychosocial functioning. Importantly, these factors are malleable, and present important foci for targeted prevention and intervention efforts [47], [48].

There remains a need to better understand the nuances of interactions between neighborhood composition and family functioning, and the impact of these on the development of young people's positive cultural identity, self-representations and onward psychosocial functioning.
